# A retrospective study on dioxins and dioxin-like polychlorinated biphenyls in milk and dairy products from the Latium region (Italy) over a 7-year study period (2011–2017)

**DOI:** 10.1007/s11356-022-20644-w

**Published:** 2022-05-14

**Authors:** Sabrina Battisti, Paola Scaramozzino, Carlo Boselli, Fabio Busico, Sesto Berretta, Marcello Sala, Bruno Neri

**Affiliations:** Istituto Zooprofilattico Sperimentale del Lazio e della Toscana ‘M. Aleandri’, Roma, Italy

**Keywords:** PCDD, PCDF, Dioxin-like PCB, Milk, Dairy products, Sampling strategy

## Abstract

This study reports the data of polychlorinated dibenzo-p-dioxins (PCDDs), -furans (PCDFs), and polychlorinated biphenyls dioxin-like (dl PCBs) measured in a total of 260 samples of the dairy supply chain collected over a period of 7 years (2011–2017) in the Latium region (Italy). Levels and average profiles of congeners were reported for each group of the analyzed dairy matrices, and any differences between different sampling strategies were considered (around likely pollutant sources or casual sampling). Of the samples, 95.4% resulted compliant with the regulated levels; only samples belonging to the “sheep bulk milk” matrix were found to be above either the action levels or the maximum levels (tot. 12 samples). Raw milk of the sheep species showed the highest averages (PCDD/F 0.248 and dl PCB 0.966 WHO TEQ pg/g of fat) compared to the milk of other species. The buffalo milk showed a content of dl PCB significantly lower (dl PCB: 0.371 WHO TEQ pg/g of fat) than the sheep milk (*p*<0.05). Dioxins were found to be superior to furans in almost all dairy products, except in the noncompliant samples where furans were higher. The OCDD was found to be the most abundant congener in almost all dairy products. This study provides a first list of reference values for background contamination of the dairy supply chain in the Latium region. These pre-existing values will be useful in all cases of environmental pollution to identify critical situations.

## Introduction

Organochlorine compounds such as polychlorinated dibenzo-p-dioxins (PCDDs), furans (PCDFs) and dioxin-like polychlorinated biphenyls (dl PCBs) are among the most studied environmental contaminants for their implication in human health and their ubiquitous character. Due to their toxic power and high persistence in the environment, these compounds are classified as persistent organic contaminants (POPs) by the Stockholm Convention (United Nations Environmental Programme (UNEP) 2019. Their widespread in the environment is mainly due to anthropogenic activities (industries, waste incineration, cement and paper factories, etc.) and the long-range transport in the atmosphere. The main route of entry into the environment of these airborne chemicals is through atmospheric deposition on the soil, from where they enter the food chain (Thiomane et al. 2018; de Lacerda 2019; Battisti et al. 2020).

The high lipophilicity of these compounds and their ability to bioaccumulate in animal and human adipose tissues is well known (González and Domingo 2021). They can cause reproductive and developmental adverse effects, damage to the immune system, and act as endocrine disruptors and carcinogens (EFSA 2018; Driesen et al. 2022). When their concentrations in tissues rise considerably, they can be harmful even after a long time from the initial exposure (Grešner et al. 2021). To mitigate the risk for consumers, the European Commission has established limit values (maximum levels and action levels) in food intended for humans and animals (Commission regulation (EC) 2006a; Commission regulation (EU) 2011; Commission regulation (EU) 2012; Commission recommendation 2014). The EU policies have led to a reduction of the assimilation of these chemicals by diet in Europe in the last years (Communication from the Commission to the Council 2001; EFSA 2018; Diletti et al. 2018).

Currently, the control of these parameters on food of animal origin is very expensive therefore sharing a large amount of data already available at the regional level becomes essential to increase knowledge and facilitate the planning of further control plans (Commission Recommendation of 16 November 2006 on the monitoring of background levels of dioxins, dioxin-like PCBs and non-dioxin-like PCBs in foodstuffs (Commission recommendation 2006/794/EC)). Even when the concentrations comply with the legal limits, the study of the data collected in the systematic control programs can provide useful information for the human exposure assessment (Scaramozzino et al. 2019).

In the event of a pollution incident, the knowledge on the pre-existing level of contamination in foodstuffs is fundamental to evaluate the real impact of a pollutant source; moreover, the background concentrations of the congeners (PCDD/Fs and dl PCBs) could provide more detailed information on the possible source of the pollutant (Esposito et al. 2009; Storelli et al. 2012; Desiato et al. 2014).

Milk has been recognized to be one of the main contributors to dietary intake of PCDD/F and dl PCB in Europe (EFSA 2018), but there is a general lack of knowledge on average levels of congeners (PCDD/Fs and dl PCBs) in the milk of different animal species, in different dairy products and on possible regional differences.

The aims of this paper are to provide the average levels of PCDD/F and dl PCB in raw milk and dairy products in the Latium region (Italy) over a sampling period of 7 years (2011–2017) to produce useful data for assessing human exposure; to show any differences in the profile of congeners between animal species, dairy products, and compliant and noncompliant samples to produce regional reference values useful for evaluation of the pollutant source impact in the chemical accident; and to analyze the spatial distribution of the noncompliant samples in relation to the sampling distribution in Latium region. Eventually, we discussed the differences with the concentrations reported by other authors in different geographical areas.

## Materials and methods

### Study area and samples

**Fig. 1:**
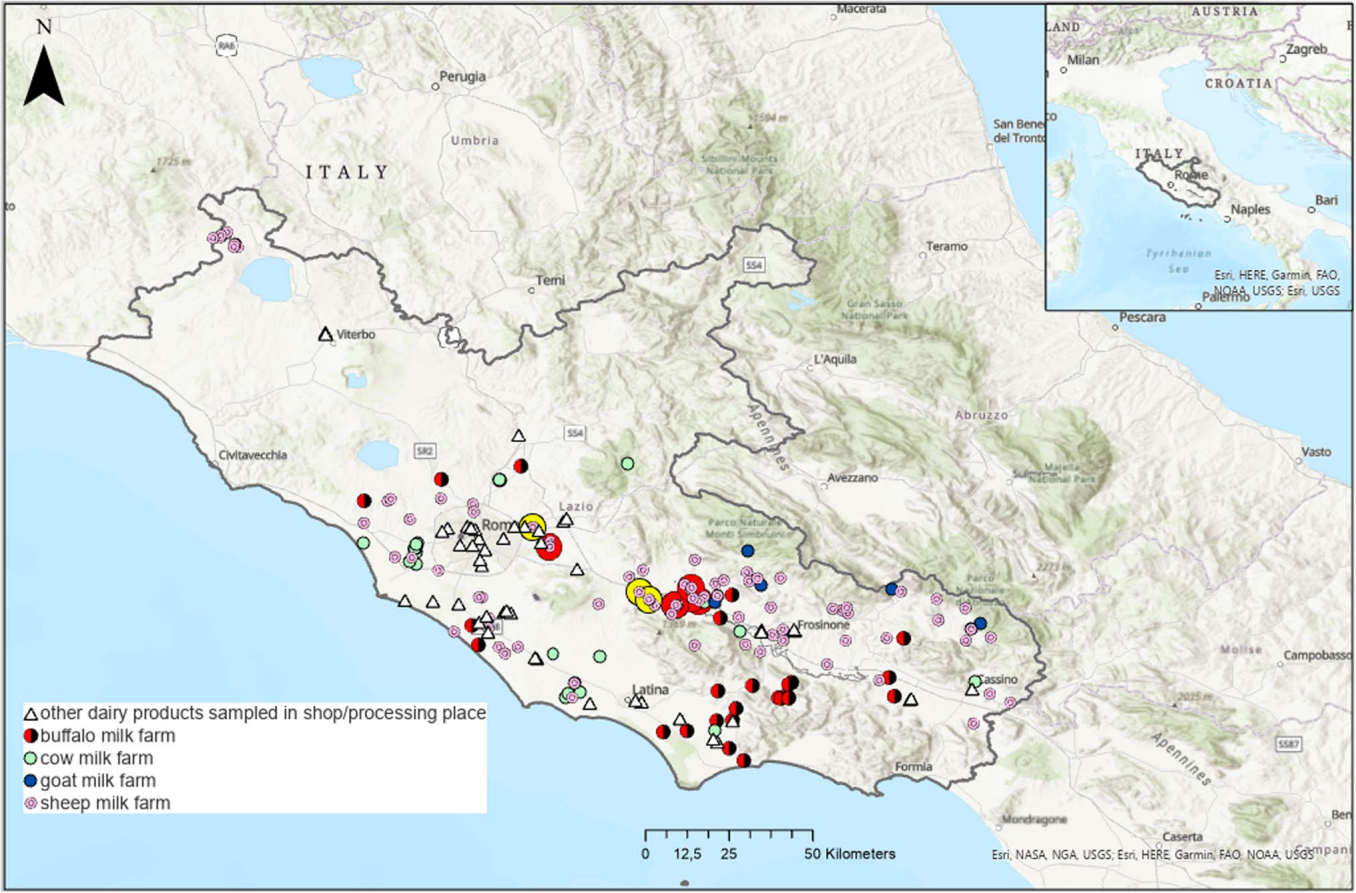
Study area and sampling sites (Latium region: 2011–2017). Geographic origin of noncompliant data (red dots>ML; yellow dots>AL), bulk milk samples (sheep, cow, buffalo, and goat milk farm), and other dairy products sampled in shop/processing place

The study reports the data on dairy products sampled for official controls in the Latium region in the period 2011–2017 and analyzed by Istituto Zooprofilattico Sperimentale del Lazio e della Toscana M. Aleandri (IZSLT) (Fig. 1). A total of 260 samples were analyzed for the concentration of PCDDs, PCDFs, and dl PCBs. The samples were belonging to the following matrices: bovine, sheep, buffalo and goat raw bulk milk (bulk milk: milk collected from the tank of a farm), drinking milk, infant formula milk, cheese, yogurt, stretched cheese/mozzarella, butter, cream, curd, fermented milk, buttermilk. In order to produce statistical indexes of contamination (means, medians, standard deviation, minimum, and maximum) and to provide the current regional background levels, all samples were grouped as follows:

- Drinking milk: whole or semi-skimmed milk subjected to heat treatment (ultrahigh temperature (UHT) milk, pasteurized milk, high-quality milk, or standard)
- Infant formula milk: powdered or follow-on liquid milk for infants
- Cheeses: soft, fresh, or matured cheeses
- Yogurt: all kinds of yogurt
- Stretched cheese/mozzarella: scamorza, mozzarella

The data of other matrices, rarely examined and whose characteristics were peculiar and not attributable to the previous groups, were also reported: butter (3 samples), cream (2), buttermilk (1), fermented milk (1), curd (1 sample). Given the small sample size, we only reported the concentrations obtained in these matrices, without drawing any conclusions.

Since samples were coming from different control plans (national monitoring plan, official food controls, food self-control, research projects, controls in potentially polluted areas), we grouped the samples into two groups to investigate the possible influence of sampling strategy on measured levels: risk-based (nearby probable pollutant source) and random sampling strategy (samples collected according to official control plans without considering possible local risk) (Regulation (EC) No 882/2004).

### Reagents and standards

All reagents and solvents used in the analysis have been tested free from contamination at the levels of interest. Columns used for the clean-up step by PowerPrep automatic system were multilayer of silica, alumina, and carbon and purchased by FMS (Fluid Management System, USA). Dichloromethane trace analysis grade, n-hexane 99% RPE grade, toluene RPE analytical grade, and ethyl acetate were purchased from Carlo Erba Reagents srl. All 13C-labeled recovery standards and clean-up and injection standard solutions were provided by CIL (Cambridge Isotope Laboratories, USA). The EPA 1613 PCDD/Fs solutions used for calibration (CS1-CS5) were provided by CIL (Cambridge Isotope Laboratories, USA). Calibration of the dlPCBs was carried out in-house by using a native analytes mixture supplied by CIL (Cambridge Isotope Laboratories, USA) and a mixture of 13C labels supplied by Wellington Laboratories.

### Analytical method

The samples were analyzed by our laboratory which is accredited by Accredia, the Italian Accreditation Body (Laboratory number 0201A), according to International Organization for Standardization UNI CEI EN ISO/IEC 17025. A total of 29 analytes, 10 PCDDs, 7 PCDFs, and 12 dlPCBs (8 mono-ortho dlPCBs and 4 non-ortho dlPCBs) were determined. The method uses high-resolution GC and high-resolution mass spectrometry (HRGC/HRMS) according to EU legislation and in agreement to US EPA (United States Environmental Protection Agency) method 1613 (US EPA 1994).

Blank and spiked samples were analyzed for each group of analysis as quality control of the trueness in the working session. Each sample was homogenized and spiked with a mixture of ^13^C-labeled standards of all dioxins and dlPCBs (isotopic dilution) and then lyophilized and extracted by means of an automatic Soxhlet extractor system (BUCHI B-811) in warm mode. The extraction was performed with an n-hexane-dichloromethane mixture with twenty extraction cycles keeping the sample at 40 °C. The extract is weighed in order to express the result per gram of fat. If the percentage of fat in the sample is less than two percent, the result is expressed in the whole sample.

The obtained extract was subjected to twice purification phases. The first phase consists in carbonizing the lipid component with a column packed with silica sulfuric acid saturated. The second phase consists of a chromatographic purification and separation of the analytes with the automatic PowerPrep system (Fluid Management System, USA) equipped with an acid-basic silica column, an alumina column, and a carbon column. Two extracts are obtained from the separation, the first containing dioxins and furans and the second containing dlPCBs.

The instrumental analysis was performed with a gas chromatographic system coupled to a high-resolution DFS (Thermo Fisher Scientific) magnetic sector mass spectrometer with a mass resolution *R*≥ 10000 (10% valley). Chromatographic separation was performed, for PCDDs and PCDFs, with a ZB-semivolatile capillary column (60 m× 0.25 mm × 0.25 μm film thickness, Phenomenex). Chromatographic separation was performed for dlPCBs with an XLB capillary column (60 m× 0.25 mm × 0.25 μm film thickness, Phenomenex).

In HRMS analysis, conditions were those reported in the US EPA 1613 method and US EPA 1668b method (2008).

Identification of congeners in the sample is considered positive if the exact masses of the monitored molecular ions and their isotopic abundance ratio match those of the congeners in the standard. The relative retention times of each congener and its ^13^C-labeled congener were used for identification.

The amount of each congener was determined by using a five-point calibration curve as reported by US EPA method 1613.

The method was validated in accordance with the Commission regulation (EC) 2006b. For the validation of the method, the following parameters were considered: the linearity (five concentration levels for each congener), specificity on twenty different samples, limit of detection (LOD) and limit of quantification (LOQ) on three concentration levels with six replicates, repeatability on three concentration levels with six replicates per level, reproducibility evaluated in two analytical sessions, trueness expressed by difference between the average value measured for each analyte in a material and its added value, expressed as a percentage of that value (Commission regulation (EC) 2006b). Trueness is compliant to validation parameters when it is into limits of ±20% of all analytes. The measurement uncertainty was determined with a metrological approach. It is ± 22% for dioxin and ± 15% for dlPCBs and is used to establish compliance of the samples.

The laboratory checks for variance every 6 months through participation in proficiency tests organized by the European Reference Laboratory for Halogenated Persistent Organic Pollutants (POPs) in Feed and Food (State Institute for Chemical and Veterinary Analysis of Food, Freiburg, Germany).

### Data analysis and statistics

To evaluate the contamination in relation to the regulated levels, the sum of PCDDs and PCDFs (hereinafter referred as PCDD/F), the sum of dl PCBs (hereinafter referred as dl PCB), and the sum of PCDD/Fs and dl PCBs (referred as PCDD/F/dl PCB) were expressed as “upper bound” (not detects posed equal to the limit of quantification: LOQ). The sums were expressed as World Health Organization (WHO) toxic equivalent (TEQ) picograms on gram lipid basis (pg/g) using the WHO toxic equivalency factors (WHO-TEFs) (Commission Regulation EU 1259/2011): pg WHO-TEQ/g fat. The results of samples with fat below 2% were expressed as pg WHO-TEQ/g.

To study the congener patterns, the levels of congeners (PCDDs, PCDFs, and dl PCBs) were analyzed in pg/g fat (for the sample with fat > 2%) or pg/g (for samples with fat < 2%), as in other studies without the transformation into TEQ (Esposito et al. 2010a, 2010b; Storelli et al. 2012; Desiato et al. 2012; Lorenzi et al. 2016; Rocha et al. 2016).

For the estimation of statistical indices (mean, median, minimum, maximum) and to test the possible differences among matrix groups, the LOQs values were used for nonquantifiable congeners both because the upper bound has to be used to assess compliance with the maximum and action levels (Commission Regulation EU 1259/2011; Commission Recommendations 2013/711/EU) and because it is more conservative, than medium and lower bound, for the purposes of health risk assessment.

All data were compared to the maximum levels (ML) of Commission Regulation EU 1259/2011 (pg WHO-TEQ/g fat), 2.5 for PCDD/F and 5.5 for PCDD/F/dl PCB, and to the action levels (AL) of Commission Recommendations 2013/711/EU and 2014/663/EU (pg WHO-TEQ/g fat): 1.75 for PCDD/F and 2.0 for dl PCB. The action levels are not applicable for food products containing < 2% fat (Commission Recommendations 2013/711/EU and Commission recommendation 2014/663/EU). For these samples, the above maximum levels must be multiplied by 0.02 (Commission Regulation EU 1259/2011).

For the purposes of this work and to study the differences in the profile in the most contaminated samples, we defined “compliant” as all samples <AL or <ML and “noncompliant” as those >AL or >ML without considering the uncertainty used to assess legal compliance. The ArcGIS software was used to map the geographical distribution of sampling and noncompliant samples in order to hypothesize possible determinants of contamination on the territory. For comparisons with other regions, we used only samples whose origin of the raw material was known and attributable to our region. This condition was met only for bulk milk matrix whose origin corresponds to the livestock farm position. The geographic coordinates of farms were extrapolated from the National Animal Registry (NAR) of the Ministry of Health of Italy. For the other dairy products, no geographical considerations were expressed since only the coordinates of the sampling points (shop, market, etc.) were available in this study and the raw materials could come from other regions.

The Stata statistical software version 16 was used to test differences and associations (*p* value<0.05) and to produce graphs. After the verification of the Gaussian distribution, the nonparametric Kruskal-Wallis equality-of-population rank test was used to found differences among animal species in bulk milk for dl PCB and PCDD/F. The Tukey post hoc test was used to test which groups were different. In order to test if the sampling strategies (risk/random) affected the concentration of pollutants in milk, we performed a Wilcoxon rank sum test, first on all data (bulk milk) and then after stratification for animal species to study the net effect of the strategy without the confounding effect due to the different distribution of animal species in the two groups. Statistical stratification was adopted in both trials to account for the confounding effect of the sampling strategy and animal species, respectively.

## Results

The sampling frequency of the analyzed matrices (tot. 260 samples) is shown in Table. 1.

**Table 1:** Frequency of analyzed samples for PCDD/F and dl PCB in the period 2011–2017 in the Latium Region (Italy

Matrices	Frequency	%
Sheep bulk milk	109	41.92
Buffalo bulk milk	44	16.92
Bovine bulk milk	26	10.00
Infant formula milk	24	9.23
Drinking milk	18	6.92
Cheese	14	5.38
Goat bulk milk	7	2.69
Yogurt	6	2.31
Stretched cheese / mozzarella	4	1.54
Butter	3	1.15
Cream	2	0.77
Curd	1	0.38
Fermented milk	1	0.38
Buttermilk	1	0.38
**Total**	**260**	**100**

The greatest level of contamination as well as the greatest variability was found in sheep milk (Table 2). Only 12 sheep milk samples (11% of the total sheep milk samples and 4,6% of the total samples) have exceeded the AL for the dl PCB (AL: 2.0 and 1.75), and among these, 6 samples have also exceeded the ML for PCDD/F or PCDD/F/dl PCB (ML: 2.5 and 5.5). Geographically, 7 of these samples came from the Sacco river valley, a well-known contaminated area (sampled with a risk-based strategy), and 5 from two farms nearby the city of Rome (sampled through random strategy).

**Table 2:** Mean, median, minimum, and maximum (upperbound values) of dl PCB, PCDD/F, and PCDD/F/dl PCB (pg WHO-TEQ/g fat; pg WHO-TEQ/g for samples with fat <2%) for different matrices (Latium region; 2011–2017)

	dl PCB					PCDD/F					PCDD/F/dl PCB				
	*Obs*	*Mean*	*Median*	*Min*	*Max*	*Obs*	*Mean*	*Median*	*Min*	*Max*	*Obs*	*Mean*	*Median*	*Min*	*Max*
Sheep bulk milk	109	0.966	0.570	0.007	11.000	109	0.248	0.146	0.010	2.410	109	1.214	0.710	0.030	11.600
Buffalo bulk milk	44	0.371	0.344	0.005	1.000	44	0.139	0.110	0.020	1.010	44	0.509	0.479	0.077	1.470
Bovine bulk milk	26	0.505	0.414	0.062	1.500	26	0.155	0.116	0.008	0.820	26	0.659	0.587	0.136	1.970
Goat bulk milk	7	0.505	0.414	0.129	1.190	7	0.284	0.239	0.053	0.700	7	0.788	0.726	0.182	1.430
Infant formula** milk	24	0.008	0.004	0.0002	0.037	24	0.004	0.003	0.0002	0.042	24	0.023	0.007	0.0005	0.282
Drinking milk	18	0.239	0.252	0.029	0.594	18	0.127	0.063	0.0007	0.760	18	0.366	0.290	0.004	1.170
Cheese	14	0.483	0.318	0.016	1.270	14	0.150	0.074	0.005	0.654	14	0.633	0.374	0.048	1.920
Yogurt	6	0.035	0.027	0.002	0.082	6	0.012	0.009	0.0009	0.032	6	0.047	0.044	0.0033	0.092
Stretched cheese/mozzarella	4	0.362	0.371	0.237	0.468	4	0.050	0.042	0.019	0.095	4	0.410	0.390	0.298	0.563
Butter	3	0.424	0.298	0.167	0.808	3	0.084	0.075	0.032	0.146	3	0.509	0.444	0.199	0.883
Cream	2	0.209	0.209	0.015	0.403	2	0.069	0.069	0.019	0.118	2	0.277	0.277	0.034	0.520
Curd	1	0.262*	-	-	-	1	0.142*	-	-	-	1	0.404*	-	-	-
Fermented milk**	1	0.0003*	-	-	-	1	0.002*	-	-	-	1	0.002*	-	-	-
Buttermilk**	1	0.002*	-	-	-	1	0.0001*	-	-	-	1	0.002*	-	-	-

*single measurement on a single sample (median, minimum, and maximum are not measurable)

**samples with fat < 2%

In order to provide reference values for PCDD, PCDF, and dl PCB, Table 2 shows the mean, median, and minimum and maximum values for all matrices

**Fig. 2:**
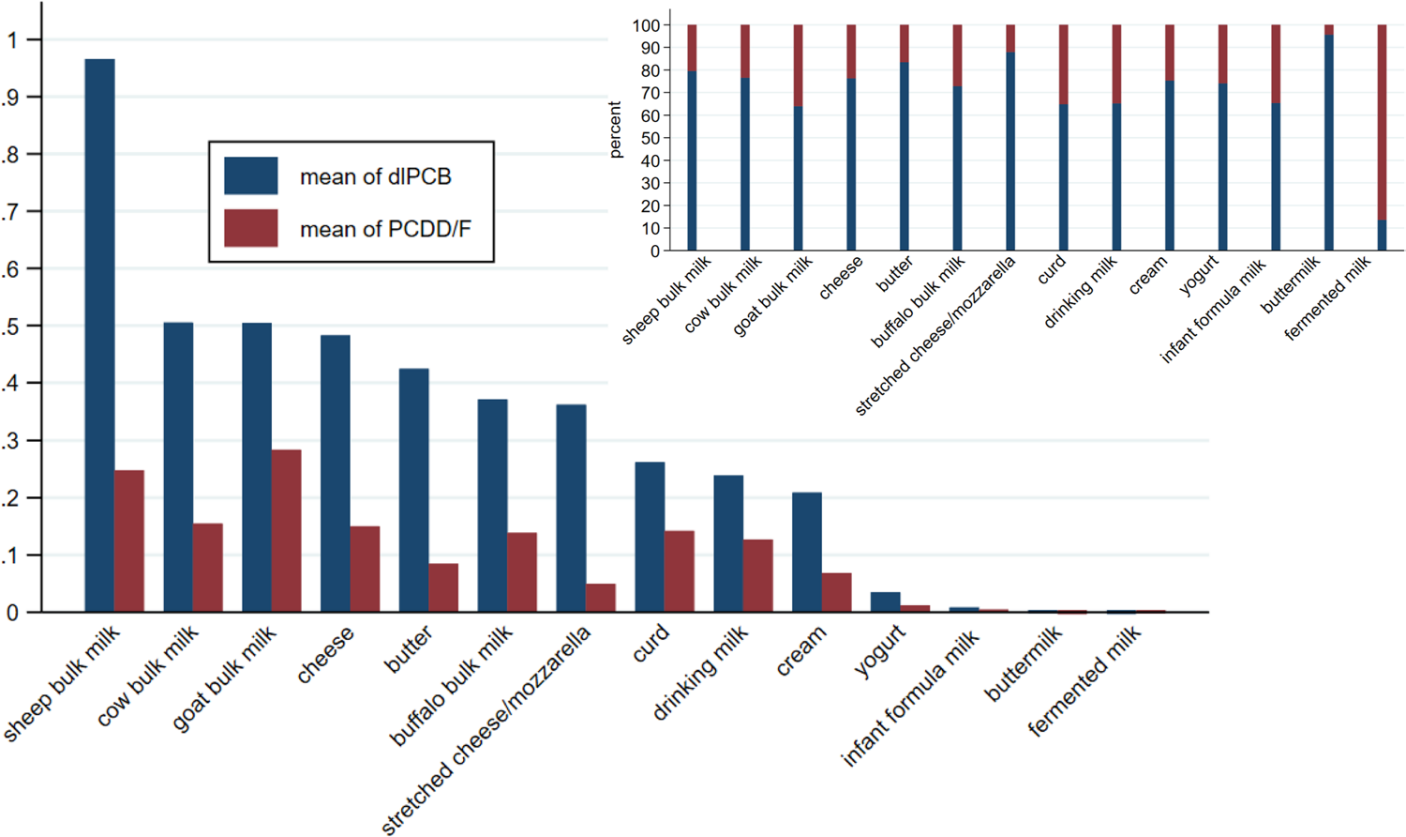
Mean values of dl PCB and PCDD/F (pg WHO-TEQ/g fat; pg WHO-TEQ/g for infant formula milk, fermented milk, buttermilk) and the average contribution (%) to the PCDD/F/dl PCB sum (percentages calculated using WHO-TEQ values) (Latium region: 2011–2017)

The buffalo bulk milk showed the lowest concentrations of dl PCB and PCDD/F compared to the milk of other animal species (cow, goat, sheep) (Table 2, Fig. 2).

The Kruskal-Wallis equality-of-population rank test found a significant difference among animal species for dl PCB and for PCDD/F/dl PCB but not for PCDD/F, showing that this difference was mainly due to the dl PCB variable. The Tukey’s test showed that the dl PCB differs significantly between buffalo and sheep milk but not among other species. The same test, performed by stratification on random strategy to take into account the confounding effect of sampling strategy (which prefers sheep samples around pollutant source), confirmed that dl PCB was significantly lower in buffalo, even when tested net of this confounder.

It was necessary to take into account this confounding effect because sheep’s milk is sampled mainly in areas at risk (risk-based strategy) due to the well-known aptitude of sheep as bioindicator. Animal distribution in the two strategies was very different in this study (risk-based strategy: 93.4% sheep bulk milk and 6.6% goat milk; random strategy: 41.6% sheep, 35.2% buffalo, 20.8% cow, 2.4% goat bulk milk (Tab. 3). Table 3 showed that in the risk-based sampling strategy, the mean values of dl PCB, PCDD/F, and PCDD/F/dl PCB were higher than in the random strategy; however, the subsequent statistical analysis showed no differences between the two groups (risk/random) net of the influence of the distribution of animal species. The higher values found in the risk strategy group were exclusively related to the confounding effect of animal distribution.

**Table 3:** Number of observations, mean, median, minimum, and maximum values of PCB dl, PCDD/F, and PCDD/F/dl PCB (pg WHO-TEQ/g fat) for raw bulk milk in the two sampling strategies (random/risk-based)

		Sampling strategy					
		Random			Risk-based		
Matrices		dl PCB	PCDD/F	PCDD/F/dl PCB	dl PCB	PCDD/F	PCDD/F/dl PCB
Buffalo milk	Obs (%)	44 (35.2%)			0 (0%)		
	Mean	0.371	0.138	0.509	.	.	.
	Median	0.343	0.11	0.478	.	.	.
	Min	0.005	0.02	0.077	.	.	.
	Max	1	1.01	1.47	.	.	.
Cow milk	Obs (%)	26 (20.8%)			0 (0%)		
	Mean	0.506	0.155	0.659	.	.	.
	Median	0.413	0.116	0.586	.	.	.
	Min	0.061	0.008	0.136	.	.	.
	Max	1.5	0.82	1.97	.	.	.
Goat milk	Obs (%)	3 (2.4%)			4 (6.6%)		
	Mean	0.384	0.282	0.666	0,95	0.286	0.88
	Median	0.39	0.092	0.726	0.442	0.263	0.801
	Min	0.129	0.053	0.182	0.306	0.182	0.488
	Max	0.634	0.7	1.09	1.19	0.432	1.43
Sheep milk	Obs (%)	52 (41.6%)			57 (93.4%)		
	Mean	0.812	0.183	0.996	1.106	0.308	1.413
	Median	0.567	0.095	0.685	0.57	0.172	0.712
	Min	0.007	0.014	0.03	0.066	0.01	0.16
	Max	5.61	1.15	6.07	11	2.41	11.6
Total	Obs (%)	125 (100%)			61 (100%)		
	Mean	0.583	0.164	0.746	1.073	0.306	1.378
	Median	0.429	0.104	0.55	0.56	0.2	0.712
	Min	0.005	0.008	0.03	0.066	0.01	0.16
	Max	5.61	1.15	6.07	11	2.41	11.6

By comparison with national averages in Diletti et al. (2018) (averages coming from 479 samples sampled in different Italian regions in the period 2013–2016, including partially also data of this study), the results in this study showed higher values (pg WHO-TEQ/g fat) for sheep (a 41,8% increase in dl PCB) and similar for buffalo milk (+ 7,8% in dl PCB and -28,9% in PCDD/F) (Tab. 4). Regarding the other species, taking into account the limitations due to the small number of samples in this study, we obtained lower values for cows (−34,4% in dl PCB and – 42,8% in PCDD/F) and goats (−60.4% in dl PCB).

**Table 4:** Average values of dl PCB, PCDD/F, and PCDD/F/dl PCB (pg WHO-TEQ/g fat) in raw bulk milk of Latium region (2011–2017) in comparison with average national values (Italy, 2013–2016) reported by Diletti et al. (2018)

		National values (Diletti et al. 2018)		Regional values in this study (Latium)	
		N° samples	Mean (WHO TEQ pg/g fat)	N° samples	Mean (WHO TEQ pg/g fat)
Buffalo milk	dl PCB	111	0.344	44	0.371
	PCDD/F		0.194		0.138
	PCDD/F/dl PCB		0.538		0.509
Cow milk	dl PCB	303	0.771	26	0.506
	PCDD/F		0.271		0.155
	PCDD/F/dl PCB		1.042		0.659
Goat milk	dl PCB	23	1.274	7	0.505
	PCDD/F		0.258		0.284
	PCDD/F/dl PCB		1.532		0.788
Sheep milk	dl PCB	142	0.681	109	0.966
	PCDD/F		0.245		0.248
	PCDD/F/dl PCB		0.926		1.214

### Congeners’ profiles

The dl PCB content resulted higher than PCDD/F in almost all dairy products, from 64 to 95.6% of the sum of PCDD/Fdl PCB, except in the single sample of fermented milk (dl PCB 13.6%) for which the lack of representativeness (only one sample) does not allow any consideration. Figure 2 shows the mean values of dl PCB, PCDD/F (WHO_TEQ), as well as the relative percentages. From a comparison between compliant and noncompliant samples, we found that dl PCB constitutes 74% of PCDD/F/dl PCB in the compliant samples and 86% in the most contaminated ones (noncompliant ones: >AL or >ML).

**Fig. 3:**
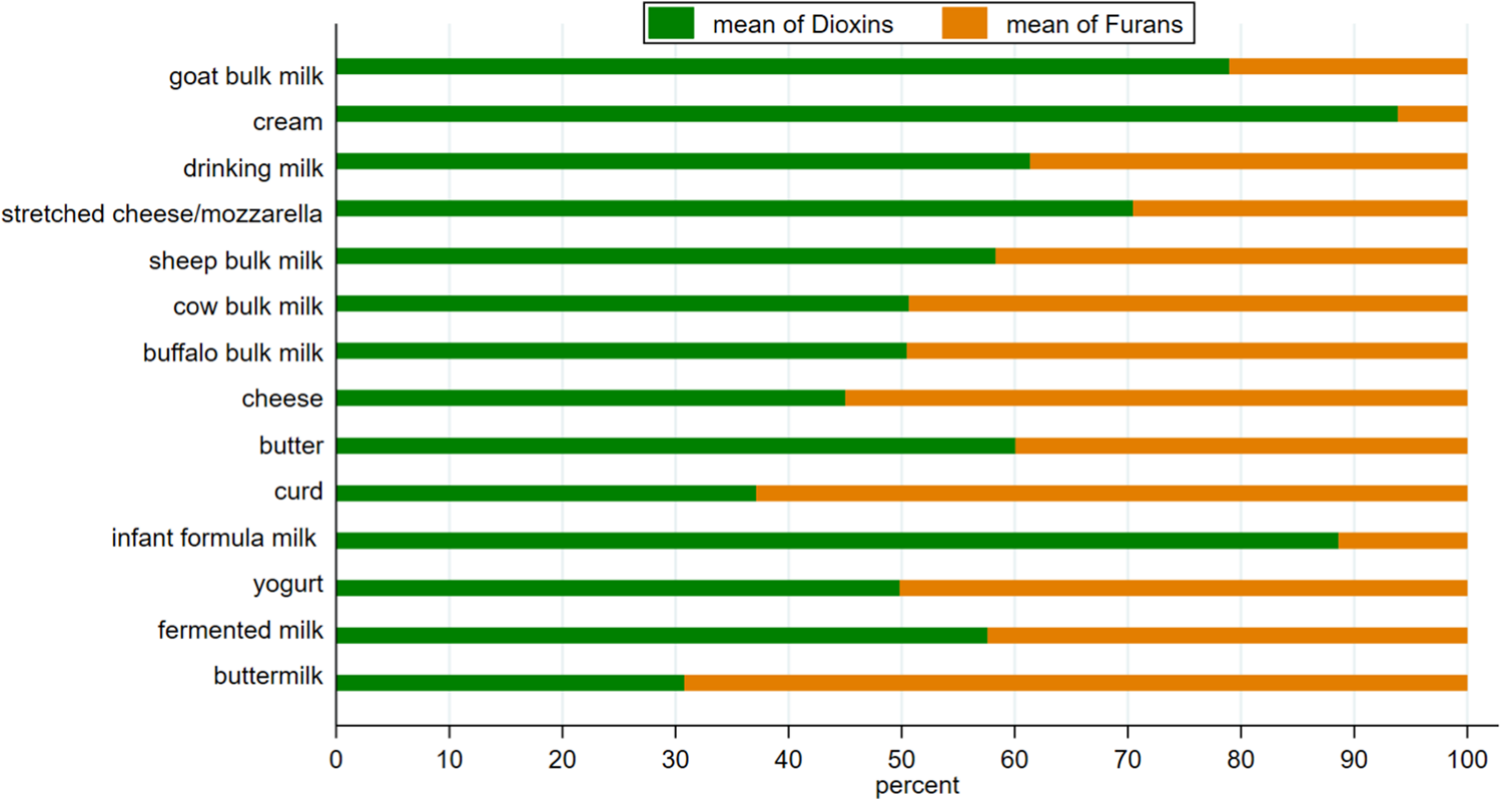
Percentage contribution (%) of PCDD and PCDF contents to the sum of PCDD/Fs (percentages calculated on values expressed as pg/g fat; pg/g for buttermilk, fermented milk, and infant formula milk) in dairy products of Latium region (2011–2017)

Regarding the contribution of dioxins (PCDD) and furans (PCDF) to the sum of PCDD/Fs, it emerged that the PCDD was prevalent: from 58 to 94% in most products (percentages measured using values expressed as pg/g fat or pg/g, depending on fat content) (Fig. 3). In cow and buffalo bulk milk, PCDD resulted in about 50% and assumed the lowest values in buttermilk (1 sample), curd (1 sample), and cheese (14 samples) (respectively: 31%, 37%, 45%) (Fig. 3). The highest percentages of PCDD were found in cream (94%) and infant formula (89%) followed by goat bulk milk (79%). In sheep milk, PCDD resulted in about 58%.

From the comparison between compliant (248 samples) and noncompliant samples (12 samples) emerged that dioxins were 61 % of the sum of PCDD/Fs in the compliant sample but only 34 % in the noncompliant ones.

**Fig. 4:**
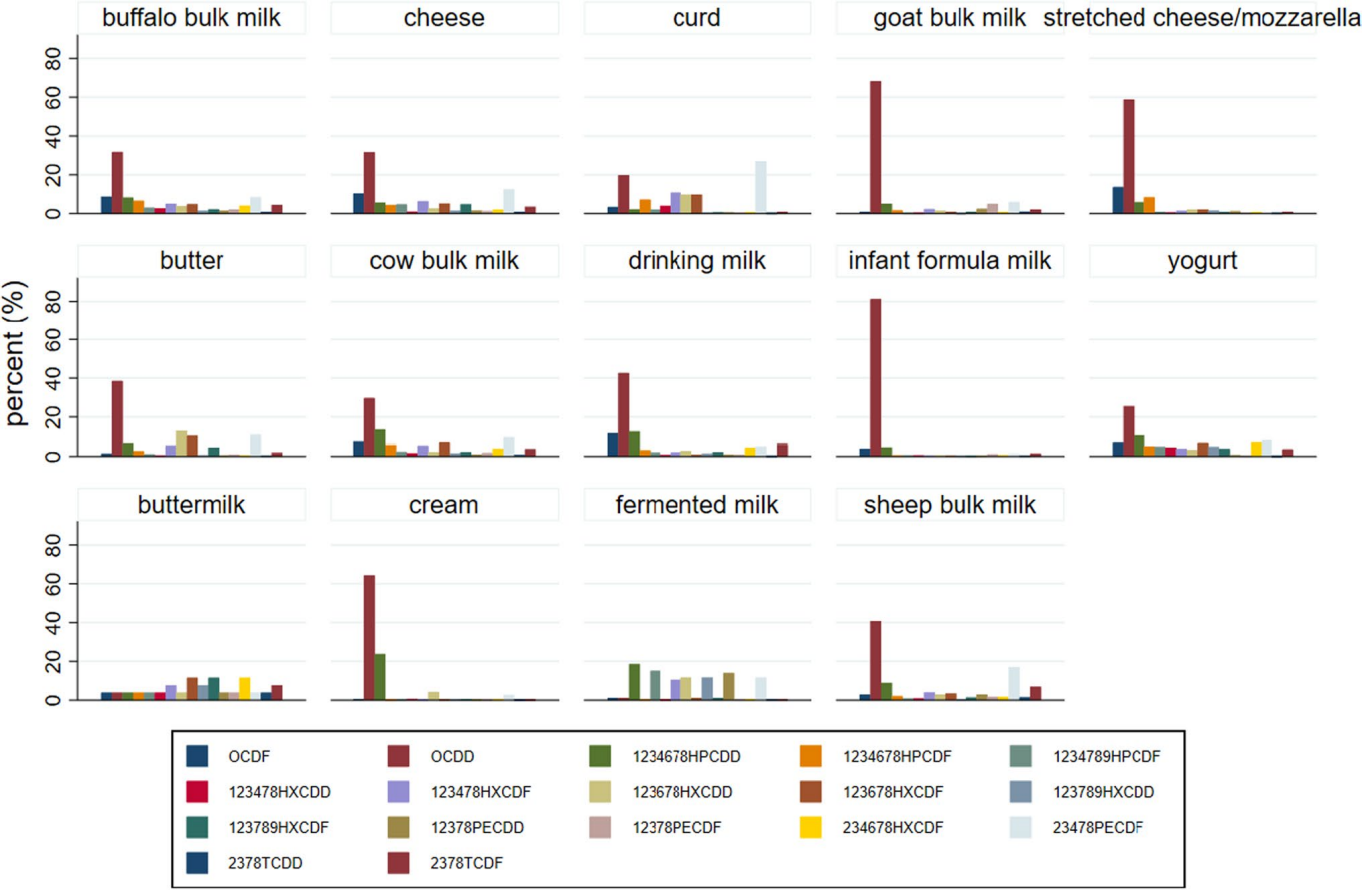
Average percentages (%) of congeners (percentages calculated on values expressed as pg/g fat or pg/g for infant formula milk, buttermilk, and fermented milk) in dairy products of the Latium region (2011–2017)

The OCDD resulted in the most abundant congener in all dairy products, with the exception of fermented milk (1 sample), buttermilk (1 sample), and curd (1 sample) for which the lack of representativeness (only one sample) and/or the very low concentrations (many congeners below the LOQ and therefore conventionally transformed into LOQ) do not allow any consideration (Fig. 4). The maximum percentage of OCDD was found in infant formula milk (81%) as well as in goat bulk milk (68%).

After the OCDD, the 2,3,4,7,8-PeCDF and 1,2,3,4,6,7,8-HpCDD or OCDF resulted in the most abundant congeners in most of the products (Table 5).

**Table 5:** Mean values of PCDDs, PCDFs, dl PCBs (pg/g fat; pg/g for samples with fat <2%) in dairy products sampled in the Latium region (period 2011–2017)

	Sheep milk	Buffalo milk	Cow milk	Goat milk	Infant formula*	Drinking milk	Cheese	Yogurt	Stretched cheese	Butter	Cream	Curd	Ferm. milk*	Buttermilk*
OCDD	0.755	0.529	0.505	3.007	0.182	0.987	0.513	0.042	1.015	0.397	1.944	0.217	0.000	0.000
1,2,3,4,6,7,8-HPCDD	0.165	0.137	0.231	0.226	0.011	0.289	0.091	0.018	0.101	0.065	0.719	0.024	0.002	0.000
1,2,3,4,7,8-HXCDD	0.016	0.046	0.030	0.016	0.001	0.018	0.017	0.008	0.009	0.006	0.020	0.044	0.000	0.000
1,2,3,6,7,8-HXCDD	0.053	0.064	0.039	0.070	0.001	0.067	0.042	0.005	0.035	0.133	0.129	0.107	0.001	0.000
1,2,3,7,8,9-HXCDD	0.009	0.025	0.027	0.007	0. .001	0.033	0.024	0.008	0.028	0.007	0.012	0.005	0.001	0.000
1,2,3,7,8-PECDD	0.054	0.025	0.017	0.106	0.001	0.020	0.026	0.001	0.021	0.006	0.010	0.007	0.001	0.000
2,3,7,8-TCDD	0.027	0.014	0.014	0.046	0.001	0.007	0.015	0.000	0.006	0.005	0.005	0.003	0.000	0.000
OCDF	0.053	0.145	0.124	0.040	0.009	0.269	0.167	0.011	0.235	0.014	0.018	0.038	0.000	0.000
1,2,3,4,6,7,8-HPCDF	0.038	0.109	0.101	0.075	0.001	0.073	0.070	0.009	0.144	0.028	0.010	0.079	0.000	0.000
1,2,3,4,7,8,9-HPCDF	0.015	0.050	0.041	0.016	0.001	0.046	0.077	0.008	0.016	0.012	0.013	0.022	0.001	0.000
1,2,3,4,7,8-HXCDF	0.073	0.084	0.093	0.101	0.001	0.048	0.104	0.006	0.024	0.057	0.012	0.118	0.001	0.000
1,2,3,6,7,8-HXCDF	0.063	0.080	0.117	0.043	0.001	0.020	0.084	0.011	0.036	0.106	0.011	0.108	0.000	0.000
1,2,3,7,8,9-HXCDF	0.025	0.038	0.040	0.041	0.001	0.053	0.077	0.006	0.013	0.047	0.014	0.007	0.000	0.000
1,2,3,7,8-PECDF	0.031	0.036	0.032	0.218	0.003	0.021	0.020	0.001	0.006	0.009	0.007	0.005	0.000	0.000
2,3,4,6,7,8-HXCDF	0.031	0.068	0.068	0.036	0.001	0.105	0.032	0.011	0.013	0.006	0.014	0.007	0.000	0.000
2,3,4,7,8-PECDF	0.314	0.139	0.162	0.261	0.003	0.117	0.201	0.013	0.007	0.111	0.079	0.297	0.001	0.000
2,3,7,8-TCDF	0.127	0.075	0.064	0.095	0.003	0.143	0.057	0.006	0.016	0.020	0.006	0.010	0.000	0.000
PCB 77	11.613	7.906	8.134	14.596	0.941	17.361	5.513	0.479	6.055	2.265	3.636	0.339	0.037	0.051
PCB 81	2.181	2.778	2.616	1.016	0.121	3.633	3.040	1.248	0.455	1.663	2.900	2.800	0.047	0.004
PCB 126	8.544	3.032	4.282	4.461	0.045	1.870	4.219	0.286	3.180	4.083	1.894	2.290	0.002	0.020
PCB 169	1.741	0.882	1.371	1.205	0.159	0.971	0.717	0.167	0.394	0.237	0.163	0.460	0.000	0.004
PCB 105	217.399	141.377	112.500	201.000	13.326	79.438	217.071	3.892	127.925	62.067	74.550	86.300	0.562	0.322
PCB 114	21.619	51.330	8.373	12.593	0.368	11.089	43.178	0.236	16.268	7.763	4.055	277.000	0.407	0.152
PCB 118	533.084	437.400	432.944	391.857	39.347	262.234	662.693	10.245	552.000	148.967	262.700	238.000	0.742	1.210
PCB 123	12.126	17.259	4.989	5.057	0.219	3.123	5.671	0.226	5.228	5.063	3.178	2.750	0.400	1.250
PCB 156	178.490	62.343	47.707	59.271	6.567	46.562	81.936	1.545	40.900	26.947	58.150	13.200	0.274	0.009
PCB 157	48.576	16.582	10.261	23.104	0.341	10.957	25.696	0.611	9.485	5.480	9.480	0.095	0.248	0.025
PCB 167	58.641	29.265	22.166	27.606	0.458	21.367	56.086	0.606	29.825	20.323	22.408	1.550	0.253	0.092
PCB189	17.424	7.374	7.171	5.958	0.113	4.282	5.122	0.675	4.003	7.123	6.950	0.100	0.005	0.015

*Samples with fat <2%

Regarding the dl PCBs, the three most represented congeners were in decreasing order: PCB 118, PCB 105, PCB 156.

In this study, only buttermilk (1 sample) and curd (1 sample) had a different pattern, but the lack of representativeness will not allow any consideration.

**Fig. 5:**
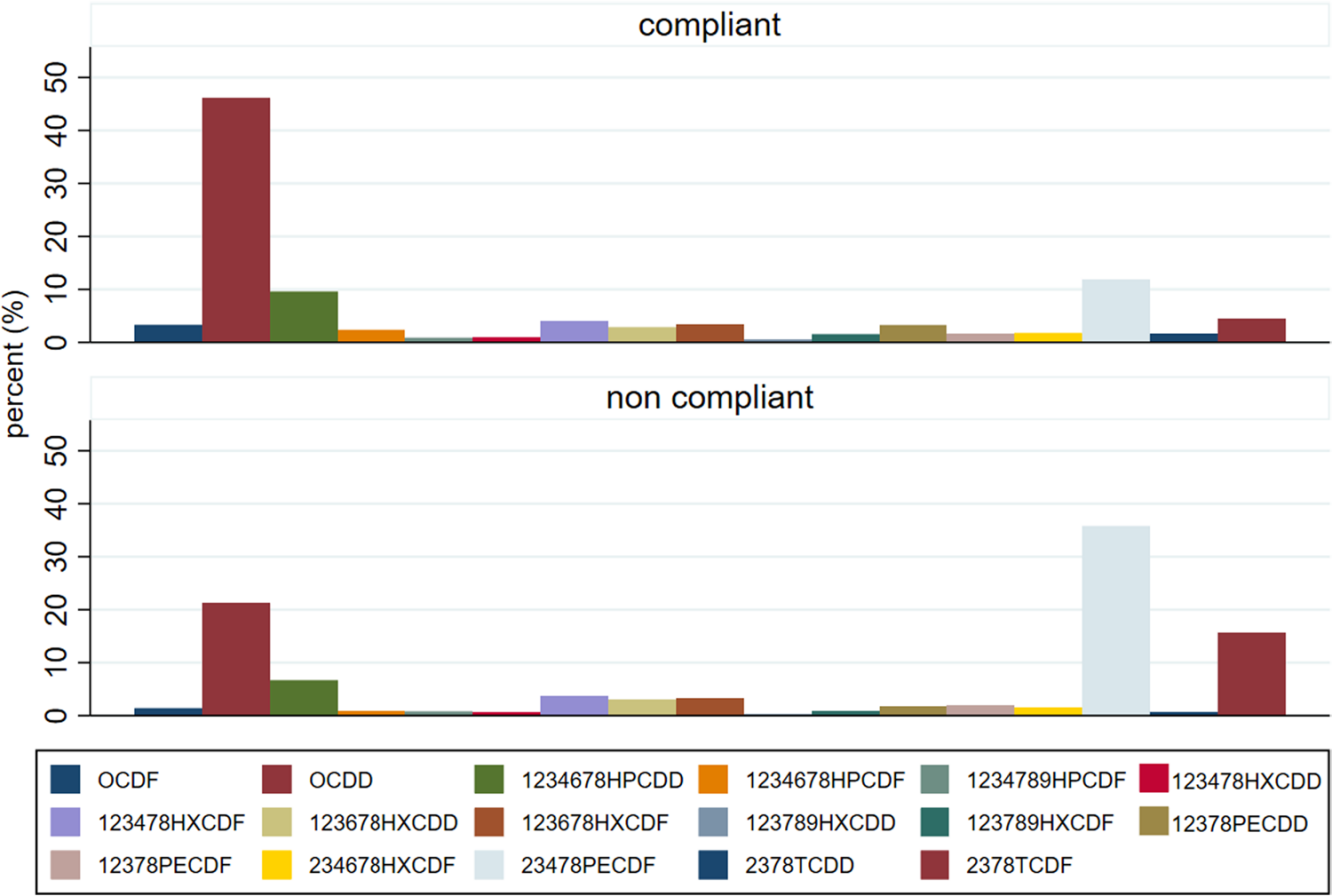
Average percentages of congeners, PCDDs, and PCDFs (percentages calculated on values expressed as pg/g fat) for compliant and noncompliant samples of sheep bulk milk (Latium region 2011–2017)

**Fig. 6:**
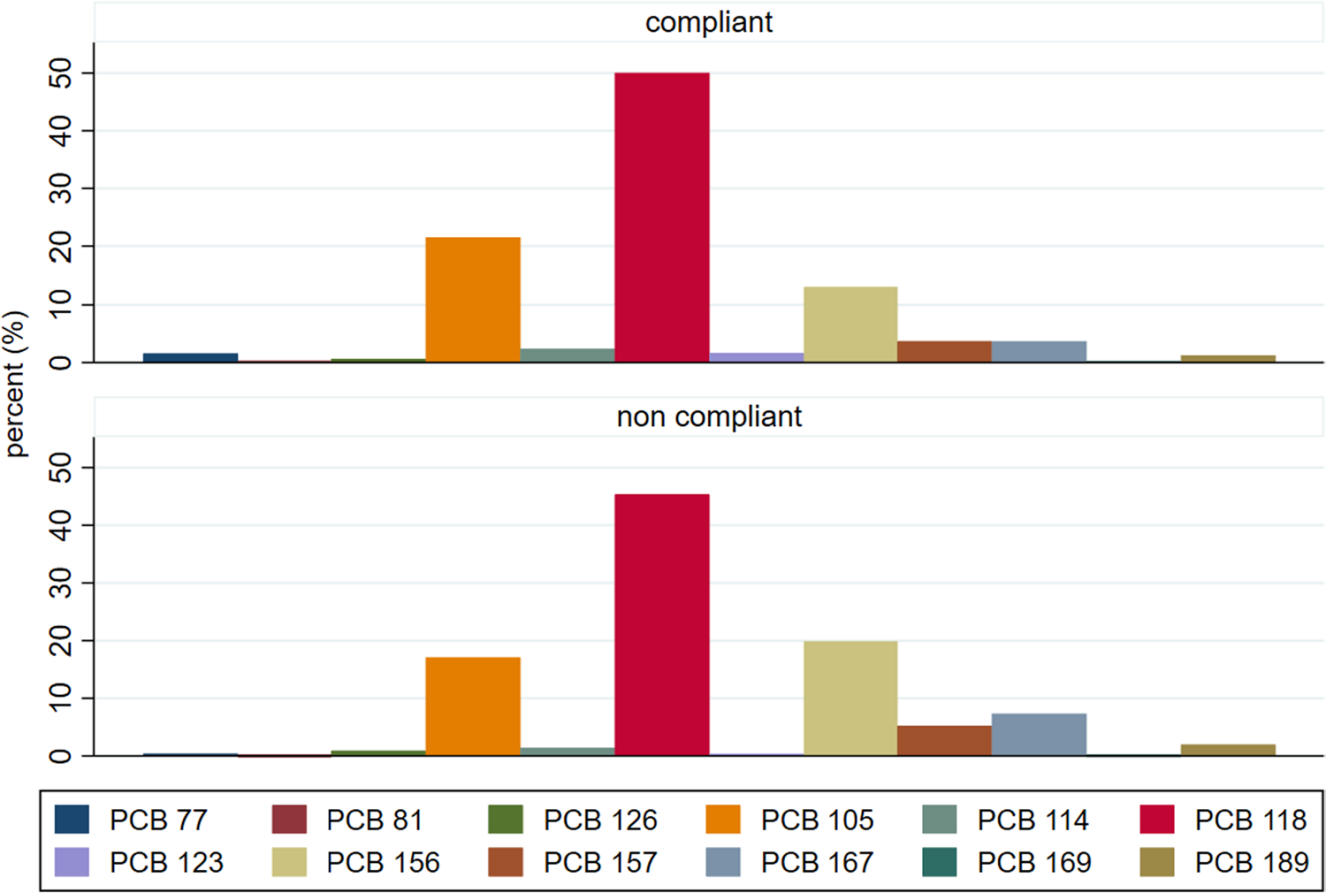
Average percentages in sheep bulk milk of dl PCBs (percentages calculated on values expressed as pg/g fat) for compliant and noncompliant (>AL: 2.0 pg/g fat) samples (Latium region 2011–2017)

Finally, regarding the sheep bulk milk, we reported congeners composition of compliant (97 samples) and noncompliant samples (>AL or >ML) to show differences in congeners (PCDD/Fs and dl PCBs) among less and most contaminated samples (Figs. 5 and 6).

As in Esposito et al. (2010a), we found that the 2,3,4,7,8-PeCDF congener becomes predominant in noncompliant samples, while the OCDD remains the most abundant in the compliant samples. In the profile of dl PCBs, we observed a small increase of the PCB 156 congener to the detriment of the PCB 105 in the noncompliant samples (Esposito et al. 2010b).

## Discussion

Given the ubiquitous nature of PCDD/Fs and dl PCBs, it is important to know their pre-existing regional levels as well as the profile of congeners in different foods to investigate possible sources of pollution or any other critical situation in the environment.

The highest concentrations (WHO-TEQ) of PCDD/F and dl PCB found in sheep milk confirmed the already well-known higher sensitivity of this matrix for environmental biomonitoring in comparison to milk of other species (Perugini et al. 2012; Scaramozzino et al. 2019). The management of sheep farming, which usually is based on grazing, could be one of the factors that favor the interaction of this species with the surrounding environment and make it more sensitive. In the Latium region, the choice of sheep’s milk in the risk-based sampling strategies (around potential sources of pollutants) resulted rightly predominant (93.4%). From a geographical point of view, the 12 noncompliant samples in this study, all belonging to sheep milk matrix, came from two confined areas, respectively, nearby the city of Rome and inside the Sacco river valley. Given the small number of highly contaminated samples and the lack of adequate environmental information, we are unable to make associations with potential polluting sources. The Sacco river valley is already known for its pollution as it has been recognized as the “site of national importance” (SIN of Sacco river) for the presence of numerous sources of pollutants (illicit waste dump, cement plants, incinerators, metal treatment plants, etc.) (Legislative Decree 2006, n. 152; Decree 2016).

The noncompliant samples (random sampled) near Rome, came from two farms which, from an analysis carried out in GIS, turned out to be close to one metal treatment plant and to a waste storage facility (data not shown, taken by European Pollutant Release and Transfer Register Regulation (EC) No 166/2006; Industrial reporting under the industrial emission directive 2010). The subsequent analysis at the farm level carried out immediately afterwards showed that the values in the milk returned below the maximum limit after a few weeks and we have no information on any in-door investigations to look for possible sources of contamination within the farms such as the adoption of incorrect practices: e.g., waste incineration, other combustion processes, etc.

No differences emerged between risk sampling strategies (around polluting sources) and random sampling, perhaps because in the period under examination, many of the sources at risk controlled by the authorities consisted of occasional large waste burning with limited effects on the environment (over time and space) demonstrated by samples slightly contaminated by PCDD/F and dl PCB.

In this study, the buffalo milk, compared to the milk of other species (sheep, goat, cow), produced the lowest average concentrations of PCDD/F and dl PCB (WHO-TEQ) as found in Diletti et al. (2018). The buffalo milk was found to be significantly less contaminated by dl PCB than sheep milk net of sampling strategy. Also Esposito et al. 2010b) indicated that on samples of buffalo milk coming from a polluted area in Caserta (Italia), the percentage contribution of dl PCB to the total WHO-TEQ (PCDD/F/dl PCB) was only one third (29%) in noncompliant samples, indicating that the contamination of buffalo milk was mainly caused by PCDD/F and not by dl PCB. Further studies are needed on buffalo species to investigate the aptitude of these animals to assimilate dl PCB since buffalo milk production in Italy (mostly used for “Mozzarella Bufala Campana”) is an important sector of economy (95% of the total buffalo milk in European Union is produced in Italy) (Manuelian et al. 2017). A selective assimilation could be due to feeding habits, farming methods, or other physiological factors, not yet proven, which may predispose buffaloes to assimilate dl PCB to a lesser extent than other herbivorous species. The buffalo breeding is in fact normally concentrated in some vacated lands (marshy areas). In this regard, it is important to highlight that the nonhomogeneous spatial sampling in our study is a limitation which does not allow to exclude that some differences are due to exposure to different environmental contexts affected by different pollutant sources (Fig. 1). From the study of the relative abundances of congeners, we found a prevalence of PCDD compared to PCDF in almost all the analyzed matrices. As the prevalence of PCDD is typical of rural areas that are not exposed to particular industrial sources of contamination, this evidence can be considered as a good indicator of the absence of particular risk situations in the Latium region (Esposito et al. 2009, 2010a). PCDF resulted predominant in the most contaminated samples (noncompliant), as in other studies (Storelli et al. 2012; Desiato et al. 2012; Esposito et al. 2010b; Bertocchi et al. 2015). In the Latium region, the OCDD was found to be the most abundant congener in most of the dairy products, followed by 2,3,4,7,8-PeCDF, 1,2,3,4,6,7,8-HpCDD, and OCDF. A limitation in the study of congeners is that when samples are poorly contaminated and many congeners are below the LOQ, the study of the relative abundances becomes inaccurate or impossible. We have analyzed the order of the most abundant congeners with the aim of making regional comparisons. The relative abundance of the three most abundant congeners (PCDD/Fs) was equal to that reported by Desiato et al. (2012) in samples not exposed to particular sources of pollution and reflecting ubiquitous contamination. This order of abundance resulted different from those reported by Storelli et al. (2012) for sheep milk in a contaminated area in Sardinia (Italy) where the three most abundant congeners were (in decreasing order) 2,3,4,7,8-PeCDF, OCDD, and OCDF (Storelli et al. 2012). They were also slightly different from those reported by Esposito et al. (2010a) for sheep and goat in the Campania region (Italy) where the three most abundant congeners were OCDD, 2,3,4,7,8-PeCDF, and 1,2,3,4,7,8-HxCDF.

The regional differences in the profiles are probably due to the presence of different industrial and anthropogenic impacts in the environment. Regarding the cow’s milk, this study reported the same decreasing order in Lorenzi et al. (2016), for the Lombardy region (Italy) (OCDD, 1,2,3,4,6,7,8-HpCDD, 2,3,4,7,8-PeCDF) but different from Emilia Romagna region (OCDD, 1,2,3,4,6,7,8-HpCDF, 2,3,4,7,8-PeCDF).

Compared to this study, Rocha et al. (2016) reported in Brazilian cow milk, a different order: OCDD, OCDF, 2,3,4,7,8-PeCDF.

Regarding the samples with the highest levels of contamination, we found that 2,3,4,7,8-PeCDF becomes more abundant than OCDD in the noncompliant sample, as found in other studies (Fig. 5) (Esposito et al. 2009; Esposito et al. 2010a; Storelli et al. 2012; Desiato et al. 2012).

Compared to the PCDD/F, the dl PCB (WHO-TEQ) was predominant in almost all dairy products (64–96% of the PCDD/F/dl PCB) as confirmed by most of the studies in several regions (Esposito et al. 2010b; Storelli et al. 2012; Desiato et al. 2012, 2014; Lorenzi et al. 2016). Regarding the dl PCBs, we found that the three most represented congeners were in decreasing order, PCB 118, PCB 105, and PCB 156, as indicated by most studies (Lorenzi et al. 2016; Esposito et al. 2010b; Brambilla et al. 2011; Desiato et al. 2012; Rocha et al. 2016). However, in the noncompliant samples (12 samples), we found a different decreasing order: PCB 118, PCB 156, PCB 105. In this regard, also in other studies, it has been reported a small increase of the PCB 156 congener to the detriment of the PCB 105 in the samples more contaminated, even when the order of the three most abundant congeners remains unchanged (Esposito et al. 2009, 2010a, 2010b).

## Conclusion

This study provides, for the first time, reference levels of congeners of PCDD/F and dl PCB in raw milk of different animal species (sheep, cow, buffalo, goat) and in different dairy products collected in the Latium region in the period 2011–2017. Although many studies report PCDD/F and dl PCB levels in the dairy supply chain, their congener-specific analysis has been rarely undertaken and compared at a regional scale. These data are important during investigations in polluted or probably polluted areas to discover any new critical issues or to identify sources responsible for pollution.

Congener profiles reflect the contamination of pasture and soil where animals graze (or feed originates) and may help to find the pollutant source even when samples comply with the legal limits. However, the transfer of PCDD/Fs and dl PCB from the environment to animals is specific for each species and congener-dependent so that the profile in milk can be very different than in soil and specific for each species and animal products or food. In this context, this study has provided a useful contribution by comparing the average profile of PCDD/Fs and dl PCBs in different animal species and foostuffs in the Latium region and comparing them with other regions’ values.
